# Parental Knowledge of Appendicitis and Its Management Options Among Children of Makkah Region, Saudi Arabia

**DOI:** 10.7759/cureus.47928

**Published:** 2023-10-29

**Authors:** Nedaa Alsulaimani, Ruba Alotaibi, Raghad Almasoudi, Renad Alamoudi, Sarah Alsharif, Ahmed Alawi

**Affiliations:** 1 Department of Medicine, Umm Al-Qura University, Makkah, SAU; 2 Pediatric Surgery, King Fahad Armed Forces Hospital, Jeddah, SAU

**Keywords:** treatment preference, patient opinion, parental knowledge, children, appendicitis

## Abstract

Background: Despite decades of studies, appendicitis in children still presents several uncertainties regarding optimal treatment.

Objectives: To assess parental understanding of appendicitis, along with its risks and treatment, and to determine attitudes to operative and non-operative treatment of uncomplicated appendicitis.

Methods: This is a cross-sectional study. The current study has targeted all parents who visited the pediatric outpatient departments or clinics at three different hospitals in Makkah and Jeddah City, including Maternity and Children Hospital (MCH) in Makkah, King Fahad Armed Force Hospital (KFAFH), and Saudi German Private Hospital (SGH) in Jeddah. Data was collected via an online Google form and was analyzed by using SPSS.

Results: A total of 408 subjects were involved in this study. The majority of them were females (74.5%); 25.5% were males. Most of the study participants aged between 25 and 34 years. Our results found that the average knowledge score of the study population was 4.1±1.81 out of 11. Only 23.5% of them had good knowledge about appendicitis. More than half of the respondents identified the appendix as a part of the digestive system and most of the study population were aware of the current treatment for appendicitis, which is surgery (80.9%). Female participants and respondents who knew someone that has been treated for appendicitis were significantly associated with a better level of knowledge about appendicitis (P-values: 0.011 and 0.033, respectively). Moreover, we found that educational level significantly influenced preference for treatment with antibiotics and surgery if appendicitis happened again (P-value: 0.049).

Conclusion: The study population had poor knowledge of appendicitis and its management options. The highlighted criteria of self-reported relevance to parents should be addressed in all appendicitis counseling and consent. We advocate for the establishment of national public awareness campaigns, as well as increased research and clinical trials. Understanding lay views of treatment alternatives and efficacy will influence future approaches to appendicitis therapy by analyzing the community's preference for emerging treatment modalities and identifying future directions for patient-centered clinical trials.

## Introduction

In Latin, appendicitis means inflammation of the appendix, combining appendix and -itis [1]. Children frequently experience abdominal pain due to acute appendicitis. It is the most frequent cause of emergency admission to pediatric surgical units. The UK treats about 10,000 cases annually [2]. The risk of having appendicitis throughout a lifetime is roughly 8%, with those between the ages of 10 and 20 being the most susceptible [3]. Despite decades of studies, appendicitis in children still presents several uncertainties regarding optimal treatment [4]. Furthermore, parents or guardians are responsible for deciding the optimal care for their children's health and well-being. Children's decisions regarding their healthcare are also influenced by their parents. Therefore, parents must possess sufficient knowledge and information to help them make decisions [5-7].

While previous similar work in this field has been undertaken, to the best of our knowledge, there has yet to be any earlier study of KSA [7]. As there may be differences in knowledge and attitudes based on culture, we aim to conduct a study at the pediatric outpatient department/clinics at the Maternity and Children Hospital (MCH) in Makkah and King Fahad Armed Force Hospital (KFAFH) in Jeddah. We will ascertain the current knowledge and understanding of parents about appendicitis along with its risks and treatment. In addition, given our current interest in the role of non-operative management of appendicitis as an alternative to surgery, we will investigate parents' attitudes toward the operative and non-operative treatment of acute uncomplicated appendicitis [8-10].

## Materials and methods

Study design

The current study is a descriptive cross-sectional study.

Study population

The current study targeted all parents who visited the pediatric outpatient departments or clinics at three different hospitals, including MCH in Makkah, KFAFH, and Saudi German Private Hospital (SGH) in Jeddah. 

Inclusion criteria

Parents (aged 16 years or over) of children (aged 18 years or younger).

Exclusion criteria

Parents who decline to participate.

Study procedure

An online questionnaire was distributed in Arabic and completed on an electronic tablet using a Google Forms platform. Our research assistants asked for voluntary participation, and IRB approval was provided.

Data collection and management

Data was collected via an online Google form that was manually distributed to the target attendance by our research assistants. The electronic responses are exported directly from the online survey platform to Microsoft Excel (Microsoft Corporation, Redmond, USA). Before analysis, the questionnaire was inspired by previous studies and adopted in the literature [7]. Some additional modifications were made to unclear or ambiguous terminology to ensure that the responses provided accurately reflect the answers the participants intended. It was validated through pilot testing with around 20 participants and was pre-tested to ensure validity. The questionnaire includes a consent form, a sociodemographic data form, previous information about appendicitis, prior knowledge of someone with appendicitis, an understanding of treatment and the risks of appendicitis, and treatment preferences, with a brief educational section before each part. A standard grading method was used for knowledge questions (10 questions): one point was given to the correct option, zero for the incorrect answer, and neutral. After data collection, a participant who scored six out of 11 was considered to have good knowledge of appendicitis. Numbers and aliases are established to ensure confidentiality. Only the authors of this study have access to the data.

Sample size determination

The minimum sample size required for this study was calculated by OpenEpi version 3.0, considering keeping the confidence interval (CI) level at 95% and assuming a 50% response rate. The sample size was calculated to be 377 participants. To enhance the sample size and account for potential data loss, we increased the sample size to 400 participants.

Statistical analysis

The obtained data was arranged in Excel sheets. After data gets extracted, it was revised, coded, and fed to statistical software IBM SPSS version 23. Descriptive statistics were obtained to summarize data, synthesize, and report the variables. Numerical data will be presented as mean±SD or as median and range according to the type of distribution of each variable. For categorical variables, percentages and frequencies were used. Chi-squared and Fisher's exact tests were used for the association between categorical variables. The P-value was considered significant if it was less than 0.05.

Ethical part and confidentiality

This study was submitted to Umm Al-Qura University and the Institutional Research Board (IRB) for approval. HAPO-02-K-012 is the registration number of the National Committee of Bioethics. The survey responses are collected anonymously. We did not obtain any identifying or private information from participants, and all answers were kept confidential.

## Results

Characteristics of the study participants

This study included a total of 408 subjects. Among those, females represented the majority (74.5%), and males were 25.5%. About one-third of the participants were 25-34 years old. Regarding the respondents' educational level, we found that most had a university degree (BA/BSc or equivalent) (61.3%). In addition, 35% of respondents reported that their oldest child was five years or less. On the other side, 33.1% mentioned that their oldest child was between 15 and 18 years (Table 1).

**Table 1:** Sociodemographic characteristics of the study participants (n=408)

Variable	Categories	Frequency	Percent
Gender	Male	104	25.5
	Female	304	74.5
Age (in years)	16-24	68	16.7
	25-34	134	32.8
	35-44	111	27.2
	45-54	60	14.7
	55-64	13	3.2
	65 or more	22	5.4
Educational level	Primary	12	2.9
	Intermediate	9	2.2
	High school	56	13.7
	Diploma	38	9.3
	University degree (BA/BSc or equivalent)	250	61.3
	Postgraduate (masters/PhD or equivalent)	43	10.5
How old is your oldest child?	5 or less	143	35
	6-10	72	17.6
	11-14	58	14.2
	15-18	135	33.1

Parental knowledge of appendicitis and its management options among children

Our study showed that the average knowledge score of the study population was 4.1±1.81 out of 11. Only 96 (23.5%) of them had good knowledge about appendicitis.

**Figure 1:**
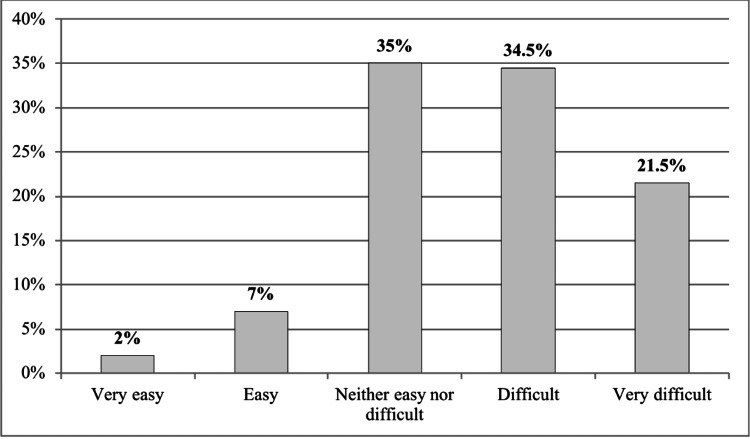
Experience of the symptoms and treatment and any complications of appendicitis among participants or those who knew them (n=200)

Our results revealed that more than half of respondents identified the appendix as a part of the digestive system (56.6%), and 30% of them were aware of the cause of appendicitis, which leads to infection and inflammation (30.9%). Moreover, 46.3% of respondents thought appendicitis was unusual in KSA (About 1 in 100). Almost half of the participants recognized the correct anatomical location of the appendix (48%), and nearly the same percentage knew someone who had been treated for appendicitis (49%). Most participants reported appendicitis in other family members (39.5%). More than one-third of respondents mentioned that the experience of symptoms and treatment and any complications of appendicitis were neither easy nor difficult (35%), nor difficult among themselves or those who knew them (34.5%) (Figure 1). 

**Figure 2:**
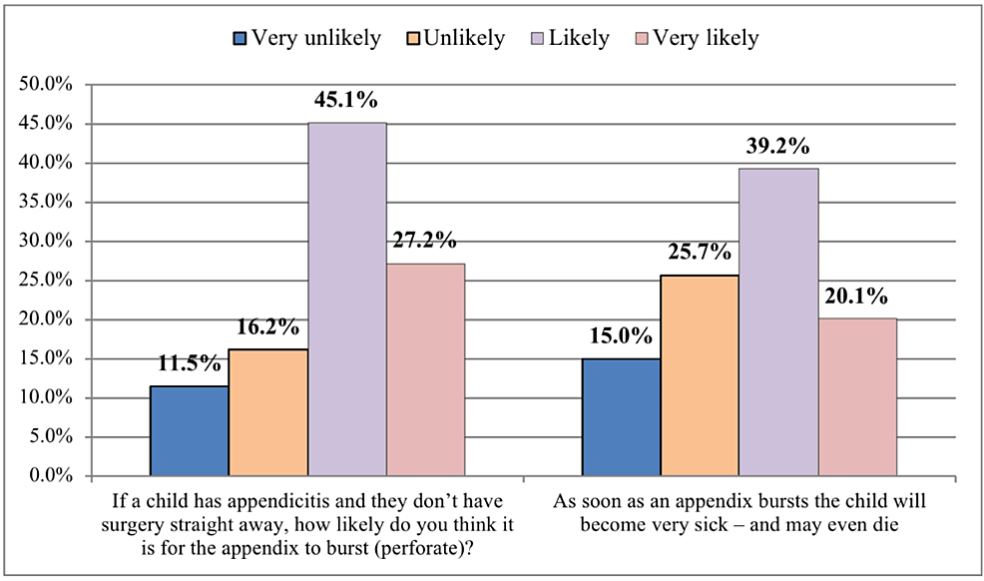
Likelihood of the appendix to burst (perforate) in case of appendicitis without surgery and the likelihood of death after a burst appendix

**Figure 3:**
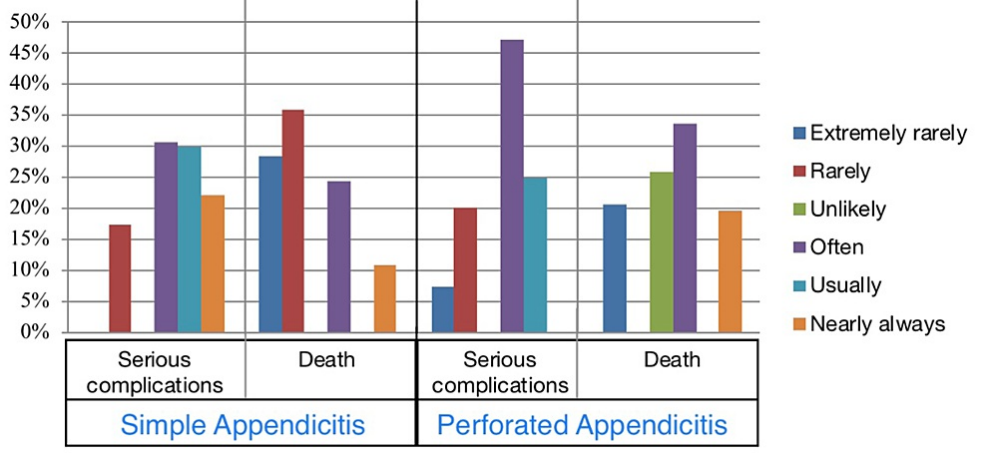
Likelihood of developing serious complications or death from appendicitis in case of simple (not burst/not perforated) appendicitis and perforated/burst appendicitis

Regarding the treatment of appendicitis, we found that most of the study population was aware of the current treatment for appendicitis, which is surgery (80.9%). Only 27.2% of participants thought that it is very likely for the appendix to perforate if the child does not have surgery immediately. Only 39.2% of respondents demonstrated that a child will likely become very sick and may even die as soon as an appendix bursts (Figure 2). Our findings showed that 30.6% of respondents reported that about one in four patients with uncomplicated appendicitis will develop serious complications, and 28.4% stated that the mortality rate of appendicitis is infrequent (under one in 1000). In regards to children with perforated/burst appendicitis, 47.3% of respondents reported that about one in four patients of uncomplicated appendicitis will develop severe complications, and 26% stated that the mortality rate of appendicitis is unlikely (about one in 10) (Figure 3).

When we assessed our participants' opinions about the treatment option for uncomplicated appendicitis, more than half of them (55.6%) preferred treatment with antibiotics (initially in the hospital and then at home) and with surgery only if the appendicitis happened again. However, 43.4% chose surgery immediately to remove the appendix (Table 2).

**Table 2:** Parental knowledge of appendicitis and its management options among children * correct answer

Variable	Categories	Frequency	Percent
What do you understand by the term appendicitis?	The appendix is a part of the digestive system*	231	56.6
	The appendix is a part of the urinary system	28	6.9
	Appendicitis arises due to eating spices and excessive spicy foods	176	43.1
	Appendicitis is caused by drinking less water	67	16.4
	Appendicitis arises due to its obstruction leading to infection and inflammation*	126	30.9
How common do you think appendicitis is in the KSA? This means the chance of developing appendicitis at any stage in a person’s life.	Very common (over 1 in 5)	15	3.7
	Common (about 1 in 10)	94	23
	Unusual (About 1 in 100)*	189	46.3
	Rare (About 1 in 1000)	51	12.5
	Extremely rare (Less than 1 in 2000)	59	14.5
Where in the body would you expect to find the appendix?	0	3	0.7
	1	5	1.2
	2	11	2.7
	3	9	2.2
	4	45	11
	5	41	10
	6	32	7.8
	7 (appendix)*	196	48
	8	30	7.4
	9	36	8.8
Do you know someone that has been treated for appendicitis?	Yes	200	49
	No	155	38
	I do not know	53	13
If yes who? (n=200)	Myself	22	11
	My child	14	7
	My parent or sibling	45	22.5
	Other family member	79	39.5
	A friend	40	20
Based on your understanding, what is the current usual treatment for appendicitis?	Pain relief and bed rest	55	13.5
	Treatment just with antibiotics	82	20.1
	Surgery (an operation to remove the appendix(*	330	80.9
If it were up to you, how would you feel about your child having the following treatments for simple appendicitis, assuming that they were equally effective:			
Treatment with antibiotics (initially in hospital and then at home) and with surgery only if the appendicitis happened again	Definitely yes	227	55.6
	Probably yes	111	27.2
	No preference	45	11
	Probably not	25	6.1
Surgery straight away to remove the appendix	Definitely yes	177	43.4
	Probably yes	101	24.8
	No preference	68	16.7
	Probably not	62	15.2

Factors associated with knowledge of appendicitis

Our results found that female participants were associated with a higher level of knowledge about appendicitis than male respondents (26.6% had good knowledge vs. 14.4%), and this association was found to be statistically significant (P-value: 0.011). Furthermore, respondents who knew someone treated for appendicitis were significantly associated with better knowledge of appendicitis than others (28%) (P-value: 0.033). Age, educational level, and oldest child's age were not significantly associated with the level of knowledge about appendicitis (P-values: 0.142, 0.060, and 0.975, respectively) (Table 3).

**Table 3:** Factors associated with knowledge of appendicitis N = number, % = percentage ^General education in the Kingdom consists of six years of primary school and three years each of intermediate and high school. *Significant P-value <0.05.

Variable	Categories	Level of knowledge		P-value
		Poor, N(%)	Good, N(%)	
Gender	Male	89 (85.6)	15 (14.4)	0.011*
	Female	223 (73.4)	81 (26.6)	
Age (in years)	16-24	54 (79.4)	14 (20.6)	0.142
	25-34	100 (74.6)	34 (25.4)	
	35-44	78 (70.3)	33 (29.7)	
	45-54	49 (81.7)	11 (18.3)	
	55-64	10 (76.9)	3 (23.1)	
	65 or more	21 (95.5)	1 (4.5)	
Educational level^	Primary	11 (91.7)	1 (8.3)	0.060
	Intermediate	9 (100)	0 (0)	
	High school	43 (76.8)	13 (23.2)	
	Diploma	33 (86.8)	5 (13.2)	
	University degree	180 (72)	70 (28)	
	Postgraduate	36 (83.7)	7 (16.3)	
How old is your oldest child?	5 or less	110 (76.9)	33 (23.1)	0.975
	6-10	55 (76.4)	17 (23.6)	
	11-14	43 (74.1)	15 (25.9)	
	15-18	104 (77)	31 (23)	
Do you know someone that has been treated for appendicitis?	Yes	144 (72)	56 (28)	0.033*
	No	121 (78.1)	34 (21.9)	
	I do not know	47 (88.7)	6 (11.3)	

Factors associated with treatment preference for a child having the following treatments for uncomplicated appendicitis, assuming that they were equally effective

When we evaluated the effect of several factors on treatment preference for a child having uncomplicated appendicitis, we found that educational level significantly influenced the choice for treatment with antibiotics and with surgery only if the appendicitis happened again (P-value: 0.049). About one-fourth of participants with a primary educational level preferred this treatment option (probably not/not: 25). In contrast, 100% of respondents who achieved an intermediate academic level chose this treatment option (definitely yes/probably yes). Our results revealed that most males and females preferred the possibility of medical treatment without any significant difference (P-value: 0.512). Age, age of the oldest child, and knowing someone that has been treated for appendicitis did not affect preference for treatment with antibiotics (initially in hospital and then at home) and with surgery only if the appendicitis happened again (P-values: 0.750, 0.369, and 0.179, respectively).

Regarding the surgical option, we found that all these factors (gender, age, educational level, age of the oldest child, and knowing someone that has been treated for appendicitis) did not significantly affect the preference for this treatment option (P-values: 0.314, 0.338, 0.726, 0.828 and 0.361, respectively) (Table 4).

**Table 4:** Factors associated with treatment preference for a child having simple appendicitis, assuming that they were equally effective ^F^P-values calculated using Fisher's exact test, and other p-values calculated using the Chi-square test. * Significant P-value <0.05.

Variable	Treatment with antibiotics (initially in hospital and then at home) and with surgery only if the appendicitis happened again			Surgery straight away to remove the appendix		
	Definitely yes/probably yes	No preference	Probably not/absolutely not	Definitely yes/probably yes	No preference	Probably not/absolutely not
Gender						
Male	89 (85.6)	11 (10.6)	4 (3.8)	75 (72.1)	18 (17.3)	11 (10.6)
Female	249 (81.9)	34 (11.2)	21 (6.9)	203 (66.8)	50 (16.4)	51 (16.8)
P value		0.512			0.314	
Age (in years)						
16-24	57 (83.8)	7 (10.3)	4 (5.9)	41 (60.3)	14 (20.6)	13 (19.1)
25-34	109 (81.3)	17 (12.7)	8 (6)	90 (67.2)	24 (17.9)	20 (14.9)
35-44	88 (79.3)	12 (10.8)	11 (9.9)	79 (71.2)	18 (16.2)	14 (12.6)
45-54	52 (86.7)	6 (10)	2 (3.3)	47 (78.3)	6 (10)	7 (11.7)
55-64	12 (92.3)	1 (7.7)	0 (0)	9 (69.2)	3 (23.1)	1 (7.7)
65 or more	20 (90.9)	2 (9.1)	0 (0)	12 (54.5)	3 (13.6)	7 (31.8)
P-value		0.750^F^			0.338	
Educational level						
Primary	8 (66.7)	1 (8.3)	3 (25)	7 (58.3)	1 (8.3)	4 (33.3)
Intermediate	9 (100)	0 (0)	0 (0)	7 (77.8)	1 (11.1)	1 (11.1)
High school	44 (78.6)	5 (8.9)	7 (12.5)	41 (73.2)	10 (17.9)	5 (8.9)
Diploma	34 (89.5)	3 (7.9)	1 (2.6)	25 (65.8)	8 (21.1)	5 (13.2)
University degree	209 (83.6)	28 (11.2)	13 (5.2)	171 (68.4)	41 (16.4)	38 (15.2)
Postgraduate	34 (79.1)	8 (18.6)	1 (2.3)	27 (62.8)	7 (16.3)	9 (20.9)
P-value		0.049*			0.726^F^	
Age of oldest child						
5 or less	115 (80.4)	16 (11.2)	12 (8.4)	92 (64.3)	26 (18.2)	25 (17.5)
6-10	59 (81.9)	9 (12.5)	4 (5.6)	52 (72.2)	10 (13.9)	10 (13.9)
11-14	50 (86.2)	3 (5.2)	5 (8.6)	38 (65.5)	12 (20.7)	8 (13.8)
15-18	114 (84.4)	17 (12.6)	4 (3)	96 (71.1)	20 (14.8)	19 (14.1)
P-value		0.369			0.828	
Know someone that has been treated for appendicitis						
Yes	159 (79.5)	24 (12)	17 (8.5)	142 (71)	33 (16.5)	25 (12.5)
No	136 (87.7)	13 (8.4)	6 (3.9)	105 (67.7)	23 (14.8)	27 (17.4)
I do not know	43 (81.1)	8 (15.1)	2 (3.8)	31 (58.5)	12 (22.6)	10 (18.9)
P-value		0.179			0.361	

## Discussion

This study aimed to assess parental understanding of appendicitis and its risks and treatment and determine attitudes to operative and non-operative treatment of uncomplicated appendicitis. Acute appendicitis is the most common surgical emergency in children. In the last decade, non-operative treatment for suspected uncomplicated appendicitis has been a hot issue [11]. Initial antibiotic treatment without surgery has been proven in studies to be just as safe and successful as surgical treatment via appendectomy [12,13].

Our findings demonstrated that the average knowledge score of the study population was 4.1±1.81 out of 11. Only 23.5% had good knowledge, whereas the highest proportion revealed poor knowledge about appendicitis (76.5%). These results were similar to another previous study, which showed a striking knowledge gap in the public’s beliefs regarding appendicitis [14]. Almost half of the participants knew someone who had been treated for appendicitis. Another study showed similar results, revealing that 49.4% reported having known someone with appendicitis [14]. In this study, almost half of the participants recognized the correct anatomical location of the appendix (48%). These findings were superior to those of a survey conducted in the UK, which revealed that one-third of respondents did not know where the appendix was [7]. The subsequent research found gaps in parental awareness, such as not knowing what appendicitis is (40% of respondents) and underestimating the incidence of appendicitis, which supports our findings. 

Regarding the treatment of appendicitis, we found that most of the study population was aware of the current treatment for appendicitis, surgery. These results go in line with another study in the UK [7]. In this study, only 27.2% of participants thought that it is very likely for the appendix to perforate if the child does not have surgery immediately. Only 39.2% of respondents demonstrated that a child will likely become very sick and may even die as soon as an appendix bursts. An earlier study found that 82% of respondents thought it was "likely" or "very likely" that the appendix would rupture if surgery were postponed, and 81% thought it would quickly lead to severe complications and death in univariate analysis, which contradicts our findings [14]. Another study in Australia found that most respondents (73%) thought appendicitis required immediate surgery, and 88% thought ruptured appendicitis was a life-threatening illness [15]. In our survey, over half of the participants agreed that appendicitis has a low fatality rate (approximately one in ten). This was greater than another study, which found that only 14% of respondents correctly identified the appendicitis fatality rate [14]. These outcome disparities are most likely related to variances in the study population's educational background.

Additionally, more than half of the participants (55.6%) preferred antibiotic treatment, and 43.4% preferred surgery to remove the appendix. This was consistent with another study, which found that two-thirds of respondents preferred surgical treatment, while 24% preferred non-operative care [16]. Furthermore, an earlier Australian investigation revealed that 52% (131/252) favored surgical management, and 48% (121/252) preferred antimicrobial treatment [15]. However, according to another survey conducted in the Netherlands, 49.2% favored antibiotic treatment for uncomplicated appendicitis, 44.5% preferred surgery, and 6.3% could not decide [17]. A recent meta-analysis found that non-operative therapies had a 97% therapeutic success rate, a 14% recurrence rate, and a comparable complication rate to surgery [8].

Furthermore, our study indicated that female participants and respondents who knew someone treated for appendicitis were significantly associated with better knowledge of appendicitis. Another prior investigation verified this, revealing that the level of expertise was considerably higher among mothers and subjects who knew at least one friend or relative who had a terrible experience with appendicitis [14]. However, a study from the UK discovered that gender, age, or previous experience with appendicitis did not affect knowledge [7]. When we looked at the effect of various characteristics on treatment preference for a child with uncomplicated appendicitis, we discovered that educational level strongly influenced the desire for antibiotic treatment. A study from the United Kingdom found no statistical difference in the proportion of mothers (41.6%) versus fathers (41.3%) who chose medical therapy, which supports our findings [14].

Our research had some limitations. First, the observational aspect of our study may limit the ability to demonstrate a causal association. Second, there could be selection bias because the number of respondents who declined survey participation should have been reported. There was a gender disparity among responders, which may have influenced our final results.

## Conclusions

The study population needed better knowledge of appendicitis and its management option. The highlighted criteria of self-reported relevance to parents should be addressed in all appendicitis counseling and consent. We advocate for the establishment of national public awareness campaigns, as well as increased research and clinical trials. Understanding lay views of treatment alternatives and efficacy will influence future approaches to appendicitis therapy by analyzing the community's preference for emerging treatment modalities and identifying future directions for patient-centered clinical trials.
